# Quantifying the direct secondary health care cost of seasonal influenza in England

**DOI:** 10.1186/s12889-020-09553-0

**Published:** 2020-09-29

**Authors:** Joe W. E. Moss, Craig Davidson, Richard Mattock, Ilana Gibbons, Stuart Mealing, Stuart Carroll

**Affiliations:** 1 York Health Economics Consortium, Enterprise House, Innovation Way, University of York, Heslington, York, YO10 5NQ UK; 2 Sanofi Pasteur UK & Ireland, Reading, UK

**Keywords:** Influenza, Burden of illness, Hospitalisation, Secondary healthcare, Vaccination, HES, Hospital episode statistics, Immunisation

## Abstract

**Background:**

The winter pressure often experienced by NHS hospitals in England is considerably contributed to by severe cases of seasonal influenza resulting in hospitalisation. The prevention planning and commissioning of the influenza vaccination programme in the UK does not always involve those who control the hospital budget. The objective of this study was to describe the direct medical costs of secondary care influenza-related hospital admissions across different age groups in England during two consecutive influenza seasons.

**Methods:**

The number of hospital admissions, length of stay, and associated costs were quantified as well as determining the primary costs of influenza-related hospitalisations. Data were extracted from the Hospital Episode Statistics (HES) database between September 2017 to March 2018 and September 2018 to March 2019 in order to incorporate the annual influenza seasons. The use of international classification of disease (ICD)-10 codes were used to identify relevant influenza hospitalisations. Healthcare Resource Group (HRG) codes were used to determine the costs of influenza-related hospitalisations.

**Results:**

During the 2017/18 and 2018/19 seasons there were 46,215 and 39,670 influenza-related hospital admissions respectively. This resulted in a hospital cost of £128,153,810 and £99,565,310 across both seasons. Results showed that those in the 65+ year group were associated with the highest hospitalisation costs and proportion of in-hospital deaths. In both influenza seasons, the HRG code WJ06 (Sepsis without Interventions) was found to be associated with the longest average length of stay and cost per admission, whereas PD14 (Paediatric Lower Respiratory Tract Disorders without Acute Bronchiolitis) had the shortest length of stay.

**Conclusion:**

This study has shown that influenza-related hospital admissions had a considerable impact on the secondary healthcare system during the 2017/18 and 2018/19 influenza seasons, before taking into account its impact on primary health care.

## Background

Seasonal influenza disease epidemics contribute considerably to winter pressures in the UK with severe cases resulting in hospitalisation due to complications. There is inadequate immunity from season to season associated with limited capacity of the influenza vaccine due to the genetic drift of the virus, which results in regular changes in the circulating strains [1, 2]. The lack of immunity from season to season stresses the need for regular re-assessment of the impact of influenza. Annual influenza epidemics are associated with approximately 290,200 to 645,800 respiratory deaths a year worldwide [3]. In the UK during the 2017–18 flu season, moderate to high levels of influenza activity were observed with co-circulation of influenza B and influenza A (H3) [4]. There were 15,969 excess deaths associated with influenza in the UK as estimated by the FluMOMO model of influenza [4]. However, laboratory confirmed influenza was associated with a total of 3175 admissions to intensive care unit/high dependency unit in England [4]. A 2016 study by Matias et al. [5] estimated the burden of influenza in the UK using weekly hospital admission and death data in combination with Public Health England’s virology reports on influenza and respiratory syncytial virus. The authors found considerable seasonal burden of illness across all age groups. Evidence suggests prevention and early intervention may be effective mechanisms for reducing the burden of influenza.

Prior to 2012, the UK vaccination policy covered groups at higher risk of severe outcomes, pregnant women, and all those aged 65 years and over [5]. However, the programme was extended in 2012 to include all children between the ages of 2 and 17 years following a recommendation by the Joint Committee on Vaccination and Immunisation (JCVI) in order to further reduce the burden and spread of influenza [6]. During the 2018 to 2019 influenza season in England, those aged 65 years and older had a vaccine uptake rate of 72.0%, whereas 48.0% of those aged 6 months to under 65 years in an at-risk group were vaccinated [7]. The lower vaccine uptake rate in those under the age of 65, and the consequence of that on secondary care resource availability for all ages, emphasises the need to assess the direct medical costs of seasonal influenza across all age groups.

Although the influenza vaccination programme in the UK is well established, those who control the hospital budget are not always involved in the prevention planning or commissioning of the programme. A recent systematic review of 27 cost of illness studies, including one from the UK, investigated the effects of influenza-related direct health care costs with a specific focus on inpatient and outpatient costs [8]. The review found that inpatient and outpatient services accounted for 43.5 and 44.5% of the total cost per episode. The study noted that global differences in the cost per influenza episode (US$19 in Korea to US$323 in Germany) may be affected by country specific characteristics [8]. The findings also highlighted that children are associated with higher costs, whereas evidence for the elderly is less conclusive. However, the study did not focus on the costs of severe influenza cases that specifically resulted in hospitalisation. Therefore, there is a need to estimate the burden of influenza on secondary healthcare. Additionally, the cost by age group needs to be considered to improve our understanding of the burden of influenza among the different age groups to support discussion around vaccination policies.

The objective of this paper is to describe the direct medical costs of secondary care influenza-related admissions across different age groups in England during the 2017/2018 and 2018/2019 influenza seasons in two ways. First, quantify the influenza-related hospital admissions, length of stay, and associated costs. Second, describe the principle costs for hospital admissions due to influenza. The costing perspective covered all mild or severe influenza admissions to secondary care including those transferred to intensive care units.

## Methods

### Data sources

Data were extracted from the Hospital Episode Statistics (HES) database for two annual influenza seasons. The data ranged from September 2017 to March 2018 and September 2018 to March 2019 in order to incorporate the annual influenza seasons. The seasonal influenza data were stratified by the following age groups: < 65, 65–74 and 75+ years in order to assess the burden of illness in both the under 65 s and the elderly. All non-elective hospital admissions, patient counts, bed days, tariff costs, and deaths were determined using international classification of disease (ICD)-10 codes. Individuals were included within the analysis if they had a primary or secondary ICD-10 code specifically related to influenza in any diagnosis field (J09–11; Table 1). However, it was not possible to extract information regarding the vaccination status for this population from the HES database. Furthermore, the number and type of comorbidities for these individuals was not assessed during this study. The inclusion and exclusion criteria of the study are summarised in Table 2.

**Table 1:** ICD-10 codes used to define influenza

ICD-10 code	Definition
J09	Influenza due to certain identified influenza viruses
J10	Influenza due to other identified influenza virus
J11	Influenza due to unidentified influenza virus

*ICD* International classification of disease

**Table 2:** Inclusion and exclusion criteria

Inclusion	Exclusion
Non-elective admissions	None
ICD-10 codes: J09–11 as a primary or secondary diagnosis	
All age groups	
Date range: September 2017 – March 2018 and September 2018 – March 2019	

Within the HES database, age is recorded in 5 year age bands based upon the recorded date of birth of the selected patients. This enabled patients to be categorised into age groups closely linked to those outlined in the UK vaccination recommendations. In order to maintain patient confidentiality, a method known as small number suppression was utilised in accordance with NHS Digital guidelines. This method involves rounding patient numbers to the nearest five to reduce the risk of individuals in small groups being identified from other published sources. The national mean length of stay was calculated from the extracted data. As the provided HES data were at an aggregated cohort level rather than at an individual patient level, it was not possible to use the median length of stay per influenza-related hospitalisation. The proportion of patients dying in hospital was estimated from those recorded as having a method of hospital discharge as “Died”.

### Costs

Hospitalisation costs were determined by using the patient’s ICD-10 codes. ICD-10 codes are not directly associated with a cost, therefore these codes are linked to a corresponding Healthcare Resource Group (HRG) code. NHS England uses HRG codes to group patient events that have been deemed to use a similar amount of healthcare resources together. HRG codes are used as tariff payments for each hospital admission or procedure. This study used the five most common HRG codes associated with the ICD-10 codes listed in Table 1 to identify the costs of non-elective influenza hospitalisations.

### Software

All analyses were performed in Microsoft Excel® (Microsoft, Redmond, Washington DC, USA).

## Results

### Influenza-related hospitalisations

In the 2017/18 influenza season, 46,215 influenza-related hospital admissions were recorded for 41,730 individual patients in England. This fell to 39,670 influenza-related hospital admissions for 35,415 patients in the 2018/2019 influenza season. This equates to 1.11 and 1.12 influenza-related hospitalisations per patient in the 2017/18 and 2018/19 influenza seasons respectively. The number of influenza-related admissions resulted in a total of 401,145 of bed days and a hospital cost of £128,153,810 with an average length of stay (LoS) of 8.68 days at an average cost of £2773 per admission in the 2017/18 season. In comparison, the 2018/19 influenza season was associated with fewer bed days (286,256) and consequently a reduced hospital cost of £99,565,310. This decrease in total number of bed days and hospital costs was expected following the reduction in the number of admissions. The average LoS for patients in the 2018/19 influenza season was 7.22 days at an average cost of £2510 per admission. The number of patients dying in hospital fell from 6.81% in the 2017/18 influenza season to 5.12% in the 2018/19 season.

### Influenza-related hospitalisations by HRG code

During the September 2017 to March 2018 and September 2018 to March 2019 influenza seasons, the top five HRG codes by influenza-related hospital admission were: WJ03 (Standard Infectious Diseases without Interventions), DZ11 (Lobar, Atypical or Viral Pneumonia, without Interventions), PD14 (Paediatric Lower Respiratory Tract Disorders without Acute Bronchiolitis), WJ06 (Sepsis without Interventions) and DZ65 (Chronic obstructive Pulmonary Disease or Bronchitis, without Interventions). Table 3 summarises the total admissions, total number of bed days and the total costs associated with each of the top five HRG codes in both influenza seasons.

**Table 3:** Top five HRG codes associated with influenza during the 2017/2018 and 2018/19 seasons

HRG Code	2017/18 influenza season			2018/19 influenza season		
	Total Admissions	Total Bed Days	Total Cost (£)	Total Admissions	Total Bed Days	Total Cost (£)
DZ11	10,865	106,905	35,753,131	8690	77,455	28,845,144
DZ65	1281	10,905	3,721,948	873	7010	2,464,886
PD14	2330	5835	3,876,050	4395	9220	6,857,201
WJ03	15,740	65,610	24,417,723	13,875	48,200	19,585,195
WJ06	2452	30,830	10,848,700	1166	14,992	5,410,835

*DZ11* Lobar, atypical or viral pneumonia, without interventions, *DZ65* Chronic obstructive pulmonary disease or bronchitis, without interventions, *PD14* Paediatric lower respiratory tract disorders without acute bronchiolitis, *WJ03* Standard infectious diseases without interventions, *WJ06* Sepsis without interventions

**Fig. 1:**
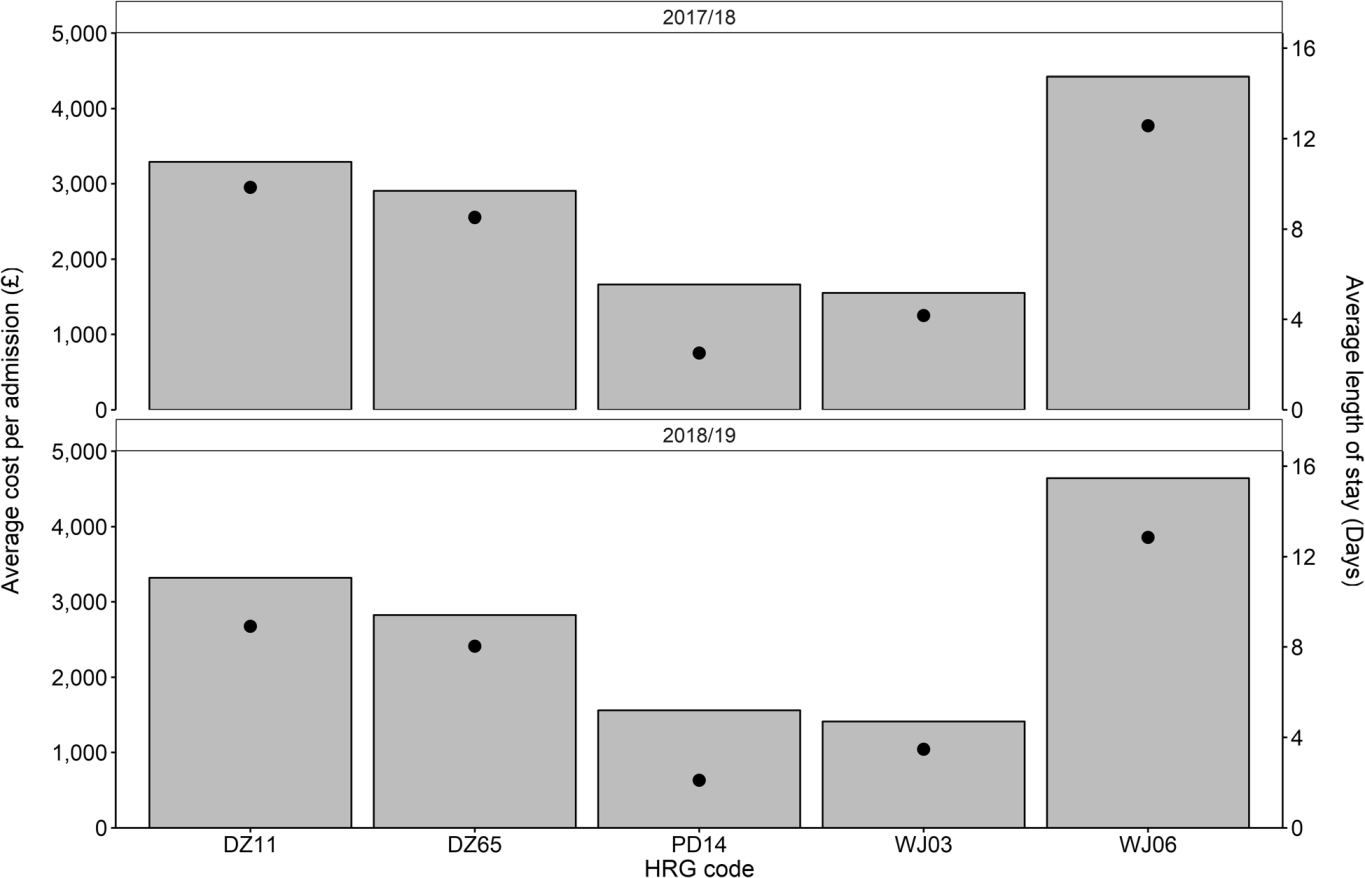
Average cost and length of stay for each admission during the 2017/18 and 2018/19 influenza seasons. The columns represent the average cost per admission, whereas the black dots indicate the average length of stay per admission for each of the top five HRG codes. The results indicate a similar trend was observed over both the September 2017 to March 2018 and the September 2018 to March 2019 influenza seasons. DZ11, Lobar, Atypical or Viral Pneumonia, without Interventions; DZ65, Chronic obstructive Pulmonary Disease or Bronchitis, without Interventions; PD14, Paediatric Lower Respiratory Tract Disorders without Acute Bronchiolitis; WJ03; Standard Infectious Diseases without Interventions; WJ06, Sepsis without Interventions

During the 2017/18 influenza season, HRG code WJ03 resulted in the largest number of influenza-related hospital admissions. However, the code was also associated with the cheapest cost per admission (~£1600) and an average LoS of 4.2 days per admission. The HRG code with the largest average LoS per admission was WJ06 at 12.9 days, approximately 28% longer than the next largest LoS (9.8 days) for DZ11 (Fig. 1). The average cost per influenza-related hospital admission for WJ06 and DZ11 was ~£4600 and ~ £3300 respectively. HRG code DZ65 was associated with the third largest average LoS at 8.5 days at an average cost of ~£2900 per admission. The shortest average LoS per admission was 2.5 days for PD14 at a cost of approximately £1700 per admission.

Similar trends were observed in the 2018/19 season, HRG code WJ06 was associated with the largest average LoS per admission at 12.9 days, approximately 44% higher than the second largest stay per admission of 8.9 days for DZ11. DZ65 was found to result in an average length of stay per admission of 8.0 days, whereas the remaining two HRG codes, WJ03 and PD14, have an average number of LoS of 3.5 and 2.1 respectively. Furthermore, the average LoS per admission was related to the average cost per admission. WJ06 was associated with an average cost of ~£4600 per admission, while DZ11 had an average cost of ~£3300 per admission. HRG codes DZ65, PD14 and WJ03 were found to have an average cost per admission of ~£2800, ~£1600 and ~ £1400 respectively.

### Influenza-related hospitalisations by age group

Further analysis of the September 2017 to March 2018 influenza season revealed that the average LoS and cost per influenza-related admission were highest in the 75+ year age group (12.55 days and ~ £3500), followed by the 65–74 year age group (9.08 days and ~ £3000). Those in the < 65 year age group were associated with an average length of stay of 5.01 days per admission with an average cost of ~£2000. The proportion of patients dying in hospital was found to increase with age, rising from 2.16% in the under 65 age group to 11.02% in the 75+ year age group.

Similar breakdown of the September 2018 to March 2019 influenza season found comparable trends to those observed in the previous season. The average LoS and cost per influenza-related admission was associated with age group, falling from 12.55 days and approximately £3700 per admission in the 75+ year age group to 4.74 days with an average cost of approximately £1900 per admission in the < 65 year age group. Those in the 65–74 year age group were found to have an average LoS of 9.04 days and an average influenza-related hospital admission cost of ~£3100. As in the previous influenza season, the proportion of patients dying in hospital was found to increase with age. The proportion of in-hospital deaths, rose from 1.83% in the under 65 age group to 11.18% in the 75+ year age group. The findings for both influenza seasons are summarised in Table 4.

**Table 4:** Influenza-related hospital outcomes by age group

Outcome	Age groups					
	2017/18 influenza season			2018/19 influenza season		
	Under 65	65–74	75+	Under 65	65–74	75+
Number of patients	17,565	7320	16,060	21,570	4975	8495
Number of admissions	19,635	8140	17,650	24,335	5605	9350
Average length of stay (days)	5.01	9.08	12.55	4.74	9.04	12.55
Average cost per admission (£)	1989.57	3023.70	3506.06	1923.60	3103.75	3675.91
Proportion dying in hospital (%)	2.16	6.20	11.01	1.83	6.51	11.18

**Fig. 2:**
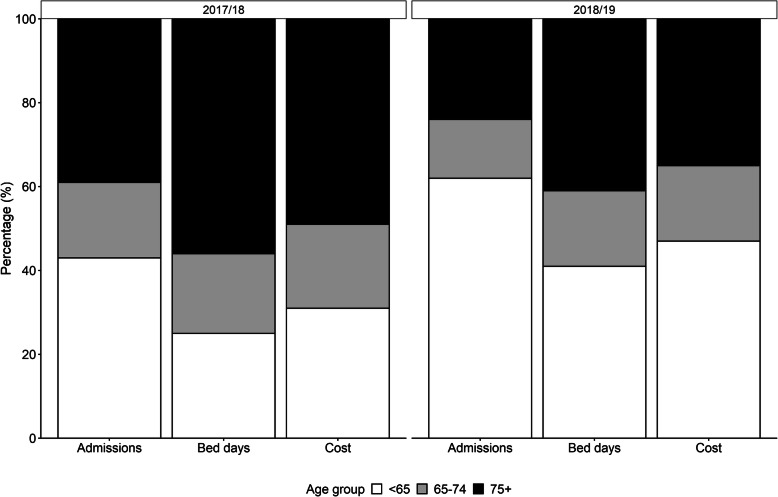
Influenza-related hospital outcomes by age group for the 2017/18 and 2018/19 influenza seasons. The proportion of patients contributing to the number of hospital admissions, bed days and hospitalisation cost were stratified by age group across both influenza seasons. The results indicate that the < 65 year age group in the 2018/19 influenza season contributed to a greater proportion of each of the three primary outcome measures compared to the 2017/18 season

Overall, the 65+ year age group accounted for 57% of all influenza-related admissions, 75% of inpatient bed days and 69% of hospital costs in England during the 2017/18 season. In contrast, during the 2018/19 influenza season, the 65+ year age group only accounted for 38% of all influenza-related admissions, 59% of inpatient bed days and 53% of hospital costs (Fig. 2).

In the 2017/18 influenza season, the HRG code WJ06 was associated with the highest LoS and cost per admission in the > 65 year age group at 14.1 days and ~ £4700 respectively. The same was observed in the 2018/19 season which found that WJ06 resulted in an average LoS of 14.9 days at an average cost of approximately £4900 per admission. Out of the top five HRG codes, PD14 contributed to 17% of the total costs in the under 65 year age group during the 2017/18 season. This rose slightly to 20% in the 2018/19 season. The longest LoS and cost per admission in the under 65 year group during the 2017/18 season was WJ06 with an average LoS of 8.9 days and an average cost of ~£3800. During the 2018/19 season, WJ06 was still associated with the longest average LoS and cost per admission in the under 65 year group. However, the average LoS and cost per admission had risen to 10.1 days and ~ £4300 respectively.

## Discussion

During both the September 2017 to March 2018 and September 2018 to March 2019 influenza seasons, there were a high number of influenza-related hospital admissions. This resulted in a considerable cost to the healthcare system in terms of secondary care. There were 41,730 and 39,670 influenza-related hospital admissions in England during the 2017/18 and 2018/19 seasons respectively. Both influenza seasons in this study show similar trends and therefore may be considered to be representative of the secondary care burden due to influenza in England over the previous two influenza seasons.

This study was performed due to the lack of evidence surrounding the impact of influenza on the secondary healthcare burden in England. The study used influenza-specific ICD-10 codes to identify all non-elective hospital admissions, patient counts, bed days, tariff costs and deaths. The HRG codes associated with the ICD-10 codes were used to calculate a cost per influenza-related hospitalisation. These costs were then stratified by the top five HRG codes and age group in order to determine the burden of influenza on secondary healthcare by age group.

The study found that HRG codes related to respiratory disease (Table 3), including pneumonia and lower respiratory tract infections collectively, were the largest contributors to total healthcare costs and influenza-related hospital admission. HRG code WJ06 (sepsis without intervention) resulted in the highest cost per admission and the longest average LoS in both the 2017/18 and 2018/19 influenza seasons. The average LoS, cost per admission and the proportion of patients dying in hospital increased with age from the under 65 year age group to the 75+ year age group.

Further revision of the current vaccination policy, for those aged 65 years and over (the greatest burden of illness on secondary healthcare resources), may further reduce the pressures and the impact of hospitalisation often facing the NHS during the winter period. This could lead to cost savings for the healthcare system. However, in vulnerable groups such as the elderly and/or the terminally ill, influenza may simply be a contributing cause of many deaths rather than the sole cause [9–11]. Influenza is known to aggravate a variety of respiratory conditions, including chronic obstructive pulmonary disease (COPD) and asthma, as well as increase the risk of myocardial infarction [12–16]. Furthermore, the influenza virus is no longer detectable in most patients when complications arise [11]. As a result, it is hard to determine the proportion of these conditions that can be attributable to influenza, especially in the elderly and/or the terminally ill [11]. Therefore, it is not certain that reducing the burden of influenza in these groups would lead to an improvement in the average quality of life expectancy [9].

Previous burden of illness studies have investigated the impact of influenza in the UK over multiple seasons by assessing retrospectively collected longitudinal data [5, 17]. Cromer et al. [17] obtained data for the 8 years prior to the A(H1N1)v pandemic in 2009 (April 2000 to March 2008). The data was assessed using a generalised linear model to determine trends over time within the data. Cromer et al. found that 37% of all annual influenza-related hospital admissions occurred in children less than 15 years old. Furthermore, this group made up 52% of all admissions amongst the non-risk groups. This finding is broadly in line with our study which found that those under the age of 65 contributed to an average of 53% of all hospital admissions in the 2017/18 and 2018/19 season. However, we cannot make a direct comparison as the age break down in our study is not as granular. The study by Matias et al. [5] modelled age-specific estimates of influenza-related hospitalisations and mortality in the UK. The study collected weekly data ranging from 1997 to 2009. Matias et al. found that on average those aged 75+ years contributed to 36% of all influenza-related hospitalisations. This is directly comparable to our study which found that 75+ year olds were responsible for 32% of all influenza-related hospitalisations in the 2017/18 and 2018/19 seasons. Furthermore, this study observed that 75+ year olds were responsible for 49% of total bed days across the two seasons, in line with previous findings by Fleming et al. [18]. The results presented within this study are in line with previous studies which have investigated the impact of influenza over multiple seasons. Therefore, our findings may be considered representative of a typical influenza season.

### Limitations

Estimating the burden of influenza-related hospital admissions is often a challenge due to the diagnostic uncertainty surrounding an accurate diagnosis of influenza as the symptoms are non-specific. Patients who present with an acute respiratory illness are not regularly assessed for virological evidence of influenza infection nor is influenza infection specified in routine databases such as hospital discharge [17]. In addition, those with a relevant ICD-10 code recorded in the secondary position may actually have hospital acquired influenza rather than influenza being the cause of admission. This study cannot attribute influenza as the sole cause of all disease burden presented due to unmeasured confounders such as the number and type of comorbidities for this population. This study was also unable to take into consideration the vaccination status of those admitted for influenza as this is not recorded within the HES database. Furthermore, the burden of influenza is often under predicted as patients frequently present with the complications of influenza rather than influenza itself. Therefore, if influenza is never suspected or tested for in a patient, their records would not be associated with an ICD-10 code listed in Table 1.

The method used within this study did not account for the number of patients who were discharged from hospital but subsequently died. Therefore, the proportion of influenza-related deaths may also be underestimated. The small number suppression technique used within this study to be compliant with NHS Digital guidelines may have resulted in a marginal over representation of the total number of influenza-related hospital admissions. Furthermore, as the LOS data were only available at a cohort level, future studies could investigate the impact of influenza on LOS at an individual patient level.

Influenza-related hospital admissions are associated with a large number of HRG codes. However, only the top five are presented in this paper. Future studies could group HRG codes together for analyses to provide a broader indication of the associated tariffs as well as include the vaccination and comorbidity status of the population.

## Conclusions

During the 2017/18 and 2018/19 influenza seasons, influenza-related hospital admissions were a substantial burden on the secondary healthcare system. The highest cost and proportion of deaths were attributed to patients age 65+ years. However, there was an increase in the proportion of costs and influenza-related hospital admissions attributable to the under 65 group during the 2018/19 season. Influenza can have a substantial impact on the healthcare system even before considering the wider impact on primary healthcare and society as a whole. Future studies assessing the burden of illness caused by influenza should include the vaccination and comorbidity status of the population to better understand the impact of these factors on the burden of illness. These studies will provide the opportunity to assess whether revision of the influenza vaccination programme may reduce winter pressures and reduce the impact on hospitals from influenza.

## Data Availability

The datasets analysed within the study are the hospital episode statistics (HES) databases which are publicly available via NHS digital. Ethical approval was not required as the release of data is controlled through the Independent Group Advising on the Release of Data (IGARD) [19] for NHS Digital. The IGARD considers all requests for dissemination of information, as defined in Section 263 of the Health & Social Care Act, through the Data Access Request Service (DARS). Access is granted to aggregate, de-identified and suppressed data. This data is licenced and provided to Health IQ (https://www.healthiq.co.uk/) who host the data and make it available to Sanofi under contract. Standard analysis of HES are made available by NHS Digital. Information on HES can be found online [20].

## Abbreviations

DZ11: Lobar, atypical or viral pneumonia, without interventions
DZ65: Chronic obstructive pulmonary disease or bronchitis, without interventions
HES: Hospital Episode Statistics
HRG: Healthcare Resource Group
ICD-10: International classification of disease-10
JCVI: Joint Committee on Vaccination and Immunisation
LoS: Length of stay
PD14: Paediatric lower respiratory tract disorders without acute bronchiolitis
WJ03: Standard infectious diseases without interventions
WJ06: Sepsis without interventions
